# The Impact of Objective and Subjective Sleep Parameters on Depressive Symptoms during Pregnancy in Women with a Mental Disorder: An Explorative Study

**DOI:** 10.3390/ijerph16091587

**Published:** 2019-05-07

**Authors:** Babette Bais, Robert Lindeboom, Leontien van Ravesteyn, Joke Tulen, Witte Hoogendijk, Mijke Lambregtse-van den Berg, Astrid Kamperman

**Affiliations:** 1 Department of Psychiatry, Erasmus University Medical Centre Rotterdam, 3015GD Rotterdam, The Netherlands; j.h.m.tulen@erasmusmc.nl (J.T.); w.hoogendijk@erasmusmc.nl (W.H.); mijke.vandenberg@erasmusmc.nl (M.L.-v.d.B.); 2 Amsterdam UMC, Academic Medical Center, Department of Clinical Epidemiology, Biostatistics and Bioinformatics, University of Amsterdam, 1100 DD Amsterdam, The Netherlands; r.lindeboom@amc.uva.nl; 3 Graduate School of Health, University of Technology Sydney, Sydney NSW 2145, Australia; l.vanravesteyn@gmail.com; 4 Department of Child and Adolescent Psychiatry/Psychology, Erasmus University Medical Centre Rotterdam, 3015GD Rotterdam, The Netherlands; 5 Epidemiological and Social Psychiatric Research Institute, Department of Psychiatry, Erasmus University Medical Centre Rotterdam, 3015GD Rotterdam, The Netherlands; a.kamperman@erasmusmc.nl

**Keywords:** sleep, circadian rhythm, depression, depressive disorder, antepartum depression, pregnancy

## Abstract

Poor sleep quality during pregnancy is associated with both antepartum and postpartum depression and adverse birth outcomes. This study evaluated both objective and subjective sleep quality and the effects on the subsequent course of antepartum depressive symptoms in psychiatric patients. This observational explorative study was embedded in an ongoing study focusing on pregnant women with a mental disorder and was performed in 18 patients (24–29 weeks pregnant). Depressive symptoms were assessed throughout pregnancy using the Edinburgh Postnatal Depression Scale (EPDS) with 5-week intervals. Sleep was assessed with actigraphy, the Pittsburgh Sleep Quality Index (PSQI) and sleep diaries at the start of the study. We studied correlations between sleep parameters and EPDS scores cross-sectionally using Spearman correlation. Next, we studied the course of antepartum EPDS scores over time per sleep parameter using generalized linear mixed modelling analysis. Objectively measured fragmentation index, total PSQI score and 4 PSQI subscales (sleep quality, sleep duration, sleep disturbances and daytime dysfunctions) were significantly correlated with EPDS scores when measured cross-sectionally at the start. Six objectively and subjectively measured sleep parameters had moderate to large effects on the course of depressive symptoms through the third trimester, but these effects were not statistically significant. More research is necessary to explore the causality of the direction between sleep problems and antepartum depressive symptoms we found in psychiatric patients.

## 1. Introduction

Depression during pregnancy is a common and high impact disease, with a prevalence of approximately 11–13% [1]. Antepartum depression has a major impact on both maternal and fetal health, as well as infant development. Children exposed to maternal depression during pregnancy have a higher risk of adverse birth outcomes and more often show cognitive, emotional and behavioral problems [2,3,4].

Many risk factors for antepartum depression have been identified, such as major life events, lack of social support, marital difficulties, low socio-economic status and an unplanned or unwanted pregnancy [5,6]. Moreover, sleep problems are common in pregnancy [7,8]: pregnant women typically show disturbed, desynchronized circadian rhythms, resulting in disturbed sleep patterns, which put them at risk for depression [9]. Poor sleep quality during pregnancy is associated with both antepartum and postpartum depressive symptoms and adverse birth outcomes [10,11,12,13,14,15,16,17], although a causal relation is difficult to prove due to the reciprocal relation of depression and sleep.

The association between poor sleep quality and mental disorders has long been recognized in the general population [18,19] and has also been shown in the peripartum period. However, many studies only studied the postpartum period [20,21] and/or only studied self-reported measures [10,12,22,23,24]. Earlier research showed that subjective sleep quality is worse than objectively measured sleep quality in pregnant women with a mental disorder [25]. A study in healthy women showed indeed that subjective sleep quality was associated with postpartum mood disturbances, but not objectively measured sleep quality [26]. However, two other studies in healthy pregnant women found that both objective and subjective sleep parameters were significantly associated with depressive symptoms in the third trimester and postpartum [15,17]. Since these three studies were executed in healthy women, these results cannot be simply extrapolated to pregnant women with a psychiatric disorder. This is relevant, since these women are at high risk for sleep problems because of not only their pregnancy, but also because of their mental problems. Based on the reciprocal relation between depression and sleep, it is relevant for clinicians and researchers to learn whether sleep problems during pregnancy are associated with depressive symptoms in psychiatric patients and whether these predict the longitudinal course of depressive symptoms.

Here, we explored both subjective and objective sleep parameters and their effects on the course of antepartum depressive symptoms in a sample of 18 psychiatric patients. We hypothesized that antepartum sleep quality is associated with antepartum depression symptom severity when assessed simultaneously during pregnancy and with its subsequent course. For this purpose, we studied both objectively measured sleep parameters from actigraphs and subjectively measured sleep parameters in relation to the course of depressive symptoms in pregnant women diagnosed with a mental disorder.

## 2. Materials and Methods

### 2.1. Participants

This study was embedded in a larger randomized controlled trial (DAPPER, NTR3015, http://www.trialregister.nl). The DAPPER study (Daycare Alternative Psychiatric Pregnant women Efficiency Research) aimed to evaluate the effectiveness of a group-based multicomponent psychotherapy intervention for pregnant women with a mental disorder, compared to individual counseling (care as usual). Both arms showed similar effects on depressive symptoms [27]. Eligible participants were pregnant women, diagnosed with a mental disorder, confirmed by the Structured Clinical Interview for DSM-IV diagnosis by one trained medical doctor [28]. A convenience subsample of participants was recruited to perform the actigraphy, PSQI and sleep diary assessments between 24 and 29 weeks of pregnancy. Participants were recruited in the second trimester, since sleep is more affected by pregnancy in the third trimester [29]. Exclusion criteria were suffering from a tremor or somatic conditions that could affect sleep, or an insufficient proficiency in Dutch.

### 2.2. Ethics

The study protocol of the randomized controlled trial was approved by the Medical Ethical Committee of the Erasmus University Medical Centre in Rotterdam, the Netherlands (registration number MEC-2009-370). Written informed consent was obtained from all participants. The present study was added to the protocol after ethical approval of the amendment.

### 2.3. Method

The following demographic information was obtained from all participants: Age, gestational age, parity, marital status, ethnicity, educational level and employment status. Educational level was dichotomized into high and low educational level, with low educational level defined as basic vocational education or less.

Objective sleep parameters were measured using the Actiwatch Actigraphy model AW4 (Cambridge Neurotechnology Ltd, Cambridge, UK). Actigraphy, the continuous assessment of activity with a watch-sized non-dominant wrist-worn recorder, is a validated technique to obtain estimates of sleep [30,31,32]. The Actiwatch measured the number of movements above a threshold setting of 20 activity counts per 60 seconds epoch, and the following indices were extracted: Total sleep time, sleep latency (time until asleep), sleep efficiency (percentage of time spent asleep while in bed) and the fragmentation index (the addition of percentage time spent moving and the percentage immobility phases of 1 minute). Lower total sleep time, higher sleep latency, lower sleep efficiency and higher fragmentation index indicate more sleep problems. Sleep data were analysed using the Actiwatch Sleep Analysis program (version 1.16, Cambridge Neurotechnology Ltd, Cambridge, UK). Participants wore the Actiwatch for 7 consecutive days and nights at the start of the study. Only the weekdays (≥3 days and nights) were used for analyses because of an increase in variability during the weekends. The precision of this assessment period was better than for 7 days [25].

Subjective sleep parameters were measured at the start with the PSQI, a structured 19-item self-rating questionnaire with 5 additional items reported by bedpartner, that assesses sleep quality and disturbances of the past month [33]. The overall sum score ranges from 0–21, with higher scores corresponding to poor sleep quality [33]. A sum score of >5 indicates poor sleep quality. The PSQI has a sensitivity and specificity of respectively 89.6% and 86.5% in distinguishing good and bad sleepers [33]. The 19 self-rated items generate 7 component scores: subjective sleep quality, sleep latency, sleep duration, habitual sleep efficiency, sleep disturbances, use of sleeping medication and daytime dysfunction [33]. Subscale scores range from 0–3, with higher component scores indicating more sleep problems [33].

Furthermore, we measured subjective sleep quality with sleep diaries kept by the participants. Participants kept a sleep diary during the week of the actigraphy assessments, which included questions on sleep time and sleep latency. A minimum of 3 weekday measurements of each participant were averaged, in analogy with the sleep parameters measured by the Actiwatch.

Depressive symptoms were assessed with the EPDS at the start of the study and subsequently at 5-week intervals until the end of pregnancy, thus 5, 10 and 15 weeks after the start. The EPDS is a structured 10-item self-report measure of depression during pregnancy [34]. Items are scored with a value 0–3, resulting in a sum score of 0–30 [34]. The EPDS was developed for the detection of postpartum depression, but has been validated for screening depression during pregnancy as well [35]. Using a cut-off of 10 or 11, depending on the trimester studied, sensitivity and specificity of the EPDS for screening antepartum depression range between respectively 70–79% and 94–97%, [35]. In the second and third trimester, a score above 10 indicates clinically relevant symptoms of depression [35].

### 2.4. Statistical Analysis

With Spearman correlation coefficients, we tested whether the various sleep parameters were correlated with EPDS scores at the start of the study. A correlation of <0.3 indicates no or a very weak relationship, of 0.3–0.5 a weak, of 0.5–0.7 a moderate and of >0.7 a strong relationship [36].

We estimated the course of antepartum EPDS scores over time using generalized linear mixed models. This is a statistical technique that, especially in the context of longitudinal designs, is robust for missing data [37], particularly in case of missing at random (MAR) and missing completely at random (MCAR). In a series of random-intercept models, we included time, the standardized sleep parameter and the time x standardized sleep parameter interaction as an effect measure of a sleep parameter on the course of antepartum EPDS scores. Subsequently, we added the standardized score of EPDS measured at the start to the model, since depression severity is an important predictor for treatment outcome [38]. For the total PSQI score, we also tested the dichotomized PSQI score, using the validated cut-off of >5 [33]. Standardized regression coefficients including the 95% Confidence Interval (CI) are reported. Regression coefficients reflect the slope of the regression line. For example, in the case of a regression coefficient of 0.15, an increase of 1 standard deviation (SD) by the sleep parameter reflects an increase of 0.15 SD on the EPDS score per measurement, thus after 5 weeks. A negative coefficient reflects a decrease in symptom severity and a positive coefficient an increase. A coefficient of <0.2 indicates a small effect, around 0.5 a moderate effect and of >0.8 a large effect [36]. By means of sensitivity analyses, we repeated our primary analysis adjusted separately one by one for medication, treatment arm, psychiatric diagnosis, age, education level, parity, work and ethnicity.

Data was checked for non-normality of distribution. Patient characteristics and sleep parameters were summarized by their median and interquartile range (IQR) for ordinal and continuous variables, since these were not normally distributed. Age and gestational age were summarized by their mean and SD. Categorical variables, such as educational level, were summarized by count and proportion. Missing data patterns were explored.

Data was analyzed using SPSS 24.0 (IBM Corporation, Chicago, IL, USA). Statistical significance was defined as *p* < 0.05.

## 3. Results

We studied 18 pregnant women. One EPDS score was missing at the start of the study, 4 at 5 weeks, 8 at 10 weeks and 13 at 15 weeks after the start. However, 5 women were at 15 weeks ≥40 weeks pregnant. Table 1 shows the demographic characteristics of the women at the start. Ten women were primarily diagnosed with depression, 5 women with generalized anxiety disorder, 2 with borderline personality disorder and 1 with bipolar disorder. Two participants drank alcohol and 7 participants smoked while pregnant. During the study, 4 participants used antidepressant medication, 1 used lithium and 1 methylphenidate.

At the start of the study, median EPDS score was 13 (IQR 8–19). Median EPDS scores were 10.5 (IQR 3.75–19.75) 5 weeks after, 13.5 (IQR 6.5–21.5) 10 weeks after and 20 (IQR 9.5–23) 15 weeks after the start.

Table 2 shows the objective and subjective measured sleep parameters at the start of the study.

Table 3 shows the correlations between the various sleep parameters and the EPDS scores at the start. A moderate relationship was found between actigraphically measured fragmentation index, PSQI sleep quality and PSQI sleep duration and EPDS score. A strong relationship was found between total PSQI score, PSQI sleep disturbances score and PSQI daytime dysfunction score and EPDS score.

Sleep efficiency, total sleep time and sleep latency were not significantly associated with depressive symptoms, both objectively and subjectively measured.

Table 4 shows the standardized regression coefficients for the course of antepartum EPDS scores for the various objectively and subjectively measured sleep parameters. None of the sleep parameters showed a significant association with the course of antepartum EPDS scores, both unadjusted and adjusted for standardized EPDS score at the start. Sensitivity analyses, in which we separately adjusted for medication, treatment arm, psychiatric diagnosis, age, education level, parity, work and ethnicity, suggested stability of the results that we found.

Though one should be careful interpreting the magnitudes of effects because of a lack of statistical significance, standardized regression coefficients differed between the studied sleep parameters. Moderate effects were found for PSQI total score, PSQI sleep duration and sleep latency measured by diary. Large effects were found for PSQI sleep quality scores and total sleep time measured by both diary and actigraphy.

Exploring missing data patterns did not show a specific selection of women with more missing EPDS assessments.

## 4. Discussion

The present study studied 18 pregnant women with a mental disorder to explore if and how objectively and subjectively measured sleep parameters would be associated with antepartum depressive symptoms. We found that actigraphically measured fragmentation index, total PSQI score (both continuous and dichotomous) and 4 PSQI subscales (sleep quality, sleep duration, sleep disturbances and daytime dysfunctions) were moderately to strongly correlated with depressive symptoms during pregnancy. We found that some sleep parameters had a notable impact on the course of antepartum depressive symptoms during the third trimester (effect size > 0.8 SD), both unadjusted and adjusted for EPDS score at the start and different separate confounders (medication, treatment, psychiatric diagnosis, age, education level, parity, work and ethnicity).

### 4.1. Cross-Sectional Findings

We found a moderate to strong correlation between total PSQI score (both continuous and dichotomized) and 4 PSQI subscales and antepartum depressive symptoms, when measured cross-sectionally. Thus, women experienced more depressive symptoms when they showed more overall sleep problems, when they reported their sleep to be of poor quality, when they reported to sleep less, when they suffered from more various sleep disturbances or when they had more problems with functioning during the day.

The finding that women who report more depressive symptoms also suffer from specific sleep problems is congruent with the relation with the total PSQI score, since this sleep parameter is an overall measure of these various underlying sleep problems. With respect to daytime dysfunctioning, it is not surprising that these symptoms correlate with depressive symptoms, since daytime dysfunctioning is a symptom of depression [28].

Two previous cross-sectional studies in peripartum women using the PSQI find associations between sleep parameters and depressive symptoms, although not consistently [23,24]. However, both these studies were executed in postpartum women, who have shown to experience more sleep problems than pregnant women [39].

We found a statistically significant negative correlation between actigraphically measured fragmentation index and antepartum depressive symptoms, when measured in the second trimester at the start of the study. Thus, women reported more depressive symptoms when the Actiwatch measured a lower fragmentation index, indicating lower mobility during sleep. This is not in line with earlier studies, where a positive relation is found between depressive symptoms and fragmented sleep [13,17,22]. Depressive symptoms are common in adults with nighttime restlessness, such as (both pregnant and non-pregnant) patients with Restless Legs Syndrome [40]. We have no explanation for this contradictory finding. We are studying a complex population where different mechanisms may be at stake. To our knowledge, sleep studies with actigraphy have not been executed earlier in a pregnant psychiatric population, so we cannot compare our findings to those from other studies. Future research should be executed to confirm our findings.

In this study, we found that the majority of the patients was suffering from sleep problems, which is common in pregnancy [7,8]. The mean PSQI score was 7.5, with 14 out of 18 patients scoring 5 or higher, an indication of clinically relevant sleep problems [33]. This score is high, but comparable to other studies peripartum. Even in studies among healthy pregnant and postpartum women without psychiatric disorders, mean PSQI scores were rather high, ranging from 6.3 to 8.3 [10,15,24,26]. A recent meta-analysis among 24 articles showed that the average PSQI score in pregnancy was 6.1 and that it increases from second to third trimester [29]. This indicates that sleep problems among pregnant women are very common. Possibly, a higher PSQI cut-off to distinguish bad sleepers is needed during pregnancy [29].

### 4.2. Longitudinal Findings

None of the sleep parameters were significantly associated with the course of antepartum depressive symptoms. However, we did find differences in standardized regression coefficients. Though not significant, probably due to the small sample size, we found large effects for PSQI sleep quality and total sleep time measured by both diary and actigraph, which may be an indication of the direction and magnitude of the effect. However, a study with more statistical power would be necessary to confirm these findings. A post-hoc power analysis revealed that a sample size of 64 women would have been needed to show significance. Another explanation for not finding any significant effects may be the fact that all women in this study were treated for their depressive symptoms, which, as a consequence, could have positively affected the course of these symptoms. At present, such studies have not been conducted yet in a population of pregnant women diagnosed with a mental disorder. Thirdly, another explanation might be the timing of assessment. Studies with antepartum measurements studied the third trimester [10,15,17,26], a timing which is comparable to our study. However, the third trimester is 12 weeks long and sleep may therefore be influenced differently along the trimester, since mothers have more sleep problems as the third trimester progresses, with women having more difficulty finding a comfortable sleeping position [7]. Adjustment for gestational age did not show that this influenced sleep in our patients differently. However, the effects of this may be small and a larger study sample may be necessary to find this. For these reasons, it is difficult to generalize our and other findings to the third trimester as a whole.

### 4.3. Strengths and Limitations

One of the strengths of this study is the use of both objective and subjective sleep parameters. Many studies rely only on self-reported measures, even though these measures often show differences with objectively measured sleep parameters [25,26]. Moreover, the PSQI and actigraphs are often used in sleep research, which makes it possible to compare the findings of this study to other studies. In addition, the EPDS is a validated tool in peripartum research worldwide and is suited to measure symptoms over a period of time. Finally, to our knowledge, this is the first study that studied the effects of both objective and subjective sleep quality on depressive symptoms in pregnant women with a psychiatric disorder.

This study also has various limitations. First, we studied a relatively small number of patients and are therefore underpowered, which makes it difficult to generalize the findings of this study. Moreover, we measured sleep quality at the start of the study and did not measure this at follow up. Studying both depressive symptoms and sleep quality longitudinally might be more suited to study how these affect each other and to understand the causality. Therefore, we do not know how sleep quality changed over time and affected depressive symptoms. Third, we measured sleep quality for one week, which is a short time period and could have been influenced by external factors. A longer time period would have limited this possibility. Fourth, our data suffer from missing EPD scores. Possibly due to their mental disorder, the participants showed less motivation to fill out all questionnaires. This could have had influence on the robustness of our findings, especially regarding the final stage of pregnancy, and which could have underestimated the effect size. However, exploring missing data patterns did not show a selection of women with more missing EPDS assessments. While our analysis did not give clear indication, we cannot rule out unmeasured factors influencing dropout. Finally, we studied a heterogeneous group of women with different mental disorders, which could have been affected differently by potential sleep problems. However, adjustment for diagnosis did not change our findings.

### 4.4. Future Directions

For future research, it would be necessary to study more patients. In addition, it would be relevant to include pregnant women without a mental disorder as a reference group. Additionally, it would be interesting to assess sleep quality at different time points, preferably before pregnancy, in all 3 trimesters and postpartum, to gain more insight in how sleep quality changes through pregnancy and how this affects depressive symptoms in the peripartum period. In a longitudinal design, the reciprocal relation between sleep and depression may be better understood.

## 5. Conclusions

We explored if and how objectively and subjectively measured sleep parameters would be associated with antepartum depressive symptoms. Various sleep parameters were significantly correlated with EPDS scores when measured cross-sectionally at the start. A number of sleep parameters had moderate to large effects on the course of depressive symptoms through the third trimester, but these effects were not statistically significant. More research is necessary to explore the causality of the direction between sleep problems and antepartum depressive symptoms we found in psychiatric patients.

## Figures and Tables

**Table 1:** Demographic characteristics of women at the start (24–29 weeks of pregnancy).

Demographics	Patients
Age in years, mean (SD)	29.5 (5.3)
Gestational age in weeks, mean (SD)	26.0 (1.7)
Parity, nulliparous	14 (78%)
Committed relationship	16 (89%)
Ethnicity, non-Western	5 (28%)
Educational level, low	13 (72%)
Unemployed	13 (72%)

**Table 2:** Distribution of objective and subjective sleep parameters at the start (24–29 weeks of pregnancy).

Sleep Parameters	Median	IQR
**Objective sleep parameters**		
Actiwatch total sleep time (hh:mm)	06:40	06:01–07:36
Actiwatch sleep latency (mm:ss)	21:46	12:03–41:59
Actiwatch sleep efficiency (%)	78.65	76.35–84.67
Actiwatch fragmentation index (%)	36.88	25.25–46.77
**Subjective sleep parameters**		
PSQI total score	7.50	4.75–11.25
PSQI sleep quality	1	1–2
PSQI sleep latency	1	0–2
PSQI sleep duration	0	0–1
PSQI sleep efficiency	1	0–3
PSQI sleep disturbances	2	1–2
PSQI sleep medication	none	none
PSQI daytime dysfunction	1	1–3
Diary total sleep time (hh:mm)	7:55	7:09–9:20
Diary sleep latency (mm:ss)	35:00	10:00–46:15

h = hour; m = minute; s = second; IQR = interquartile range.

**Table 3:** Correlations between objective and subjective sleep parameters and EPDS scores when measured cross-sectionally at the start (24–29 weeks of pregnancy).

Sleep Parameters	r (SE)
**Objective sleep parameters**	
Actiwatch fragmentation index	−0.51 * (0.22)
Actiwatch total sleep time	0.14 (0.25)
Actiwatch sleep efficiency	0.41 (0.20)
Actiwatch sleep latency	0.40 (0.26)
**Subjective sleep parameters**	
PSQI total score	0.79 *** (0.15)
PSQI total score, dichotomized	0.78 *** (0.10)
PSQI sleep quality	0.68 ** (0.16)
PSQI sleep latency	0.35 (0.26)
PSQI sleep duration	0.53 * (0.23)
PSQI sleep efficiency	−0.38 (0.22)
PSQI sleep disturbances	0.82 *** (0.13)
PSQI sleep medication	none
PSQI daytime dysfunction	0.82 ** (0.12)
Diary total sleep time	−0.16 (0.26)
Diary sleep latency	0.28 (0.29)

SE = standard error; * *p* < 0.05; ** *p* < 0.01; *** *p* < 0.001.

**Table 4:** Effects of objective and subjective sleep parameters at the start (24–29 weeks of pregnancy) on the course of depressive symptoms through the third trimester.

Sleep Parameters	Standardized β (95% CI)	Standardized β_adj_ (95% CI) ^
**Objective sleep parameters**	
Fragmentation index	−0.56 (−2.39–1.28)	−0.28 (−2.01–1.47)
Total sleep time	1.23 (−0.42–2.89)	1.25 (−0.47–2.97)
Sleep efficiency	−0.03 (−1.87–1.81)	−0.36 (−2.12–1.39)
Sleep latency	−0.27 (−1.60–1.06)	−0.24 (−1.54–1.06)
**Subjective sleep parameters**		
PSQI total score	−0.46 (−2.29–1.38)	−0.48 (−2.12–1.17)
PSQI total score, dichotomized	−0.45 (−2.10–1.20)	−0.26 (−1.84–1.31)
PSQI sleep quality	1.73 (−2.78–6.24)	1.99 (−1.01–4.98)
PSQI sleep latency	0.41 (−1.12–1.95)	0.35 (−1.11–1.81)
PSQI sleep duration	−0.53 (−3.96–2.90)	0.72 (−1.35–2.78)
PSQI sleep efficiency	−0.19 (−1.82–1.44)	0.11 (−1.37–1.59)
PSQI sleep disturbances	0.27 (−3.26–3.79)	0.48 (−2.25–3.20)
PSQI sleep medication	none	none
PSQI daytime dysfunction	−0.36 (−4.33–3.62)	−0.15 (−2.51–2.22)
Diary total sleep time	1.12 (−0.31–2.55)	1.25 (−0.33–2.83)
Diary sleep latency	0.70 (−0.83–2.22)	0.75 (−0.84–2.33)

^ Adjusted for standardized EPDS score at the start.

## References

[1] Woody C., Ferrari A., Siskind D., Whiteford H., Harris M. (2017). A systematic review and meta-regression of the prevalence and incidence of perinatal depression. J. Affect. Disord..

[2] Grote N.K., Bridge J.A., Gavin A.R., Melville J.L., Iyengar S., Katon W.J. (2010). A Meta-analysis of Depression During Pregnancy and the Risk of Preterm Birth, Low Birth Weight, and Intrauterine Growth Restriction. Arch. Gen. Psychiatry.

[3] Jarde A., Morais M., Kingston D., Giallo R., MacQueen G.M., Giglia L., Beyene J., Wang Y., McDonald S.D. (2016). Neonatal outcomes in women with untreated antenatal depression compared with women without depression: a systematic review and meta-analysis. JAMA Psychiatry.

[4] Talge N.M., Neal C., Glover V. (2007). Early Stress, Translational Research and Prevention Science Network: Fetal and Neonatal Experience on Child and Adolescent Mental Health. Antenatal maternal stress and long-term effects on child neurodevelopment: how and why?. J. Child Psychol. Psychiatry.

[5] Lancaster C.A., Gold K.J., Flynn H.A., Yoo H., Marcus S.M., Davis M.M. (2010). Risk factors for depressive symptoms during pregnancy: a systematic review. Am. J. Obstet. Gynecol..

[6] Räisänen S., Lehto S.M., Nielsen H.S., Gissler M., Kramer M.R., Heinonen S. (2014). Risk factors for and perinatal outcomes of major depression during pregnancy: a population-based analysis during 2002–2010 in Finland. BMJ Open.

[7] Mindell J.A., Jacobson B.J. (2000). Sleep Disturbances During Pregnancy. J. Obstet. Gynecol. Neonatal Nurs..

[8] Mindell J.A., Cook R.A., Nikolovski J. (2015). Sleep patterns and sleep disturbances across pregnancy. Sleep Med..

[9] Facco F.L., Kramer J., Ho K.H., Zee P.C., Grobman W.A. (2010). Sleep Disturbances in Pregnancy. Obstet. Gynecol..

[10] Kamysheva E., Skouteris H., Wertheim E.H., Paxton S.J., Milgrom J. (2010). A prospective investigation of the relationships among sleep quality, physical symptoms, and depressive symptoms during pregnancy. J. Affect. Disord..

[11] Palagini L., Gemignani A., Banti S., Manconi M., Mauri M., Riemann D. (2014). Chronic sleep loss during pregnancy as a determinant of stress: impact on pregnancy outcome. Sleep Med..

[12] Lewis B.A., Gjerdingen D., Schuver K., Avery M., Marcus B.H. (2018). The effect of sleep pattern changes on postpartum depressive symptoms. BMC Women’s Health.

[13] Okun M.L., Kiewra K., Luther J.F., Wisniewski S.R., Wisner K.L. (2011). Sleep Disturbances in Depressed and Non-Depressed Pregnant Women. Depress. Anxiety.

[14] Okun M.L., Luther J.F., Wisniewski S.R., Sit D., Prairie B.A., Wisner K.L. (2012). Disturbed Sleep, a Novel Risk Factor for Preterm Birth?. J. Women’s Health.

[15] Calcagni S.C., Bei B., Milgrom J., Trinder J. (2012). The Relationship Between Sleep and Mood in First-Time and Experienced Mothers. Behav. Sleep Med..

[16] Lee K.A., Gay C.L. (2004). Sleep in late pregnancy predicts length of labor and type of delivery. Am. J. Obstet. Gynecol..

[17] Park E.M., Meltzer-Brody S., Stickgold R. (2013). Poor sleep maintenance and subjective sleep quality are associated with postpartum maternal depression symptom severity. Arch. Women’s Health.

[18] Lee E.K., Douglass A.B. (2010). Sleep in Psychiatric Disorders: Where are We Now?. Can. J. Psychiatry.

[19] Regen W., Nanovska S., Spiegelhalder K., Baglioni C., Riemann D. (2013). Comorbid Sleep Disorders in Neuropsychiatric Disorders Across the Life Cycle. Curr. Psychiatry Rep..

[20] Posmontier B. (2008). Sleep Quality in Women with and without Postpartum Depression. J. Obstet. Gynecol. Neonatal Nurs..

[21] Tsai S.-Y., Thomas K.A. (2012). Sleep disturbances and depressive symptoms in healthy postpartum women: A pilot study. Nurs. Health.

[22] Goyal D., Gay C., Lee K. (2009). Fragmented maternal sleep is more strongly correlated with depressive symptoms than infant temperament at three months postpartum. Arch. Women’s Health.

[23] Huang C.-M., Carter P.A., Guo J.-L. (2004). A Comparison of Sleep and Daytime Sleepiness in Depressed and Non-Depressed Mothers During the Early Postpartum Period. J. Nurs..

[24] Dørheim S.K., Eberhard-Gran M., Bjorvatn B., Bondevik G.T. (2009). Sleep and Depression in Postpartum Women: A Population-Based Study. Sleep.

[25] Van Ravesteyn L.M., Tulen J.H., Kamperman A.M., Raats M.E., Schneider A.T., Birnie E., Steegers E.A., Hoogendijk W.J., Tiemeier H.W., Berg M.P.L.D. (2014). Perceived Sleep Quality Is Worse Than Objective Parameters of Sleep in Pregnant Women with a Mental Disorder. J. Clin. Sleep Med..

[26] Bei B., Ericksen J., Trinder J., Milgrom J. (2010). Subjective Perception of Sleep, but not its Objective Quality, is Associated with Immediate Postpartum Mood Disturbances in Healthy Women. Sleep.

[27] Van Ravesteyn L.M., Kamperman A.M., Schneider T.A., Raats M.E., Steegers E.A., Tiemeier H., Hoogendijk W.J., Berg M.P.L.-V.D. (2018). Group-based multicomponent treatment to reduce depressive symptoms in women with co-morbid psychiatric and psychosocial problems during pregnancy: A randomized controlled trial. J. Affect. Disord..

[28] First M.B., Gibbon M., Spitzer R.L. (2002). User’s Guide for the SCID-I. Structured Clinical Interview for DSM-IV TR Axis I Disorders (Research Version).

[29] Sedov I.D., Cameron E.E., Madigan S., Tomfohr-Madsen L.M. (2018). Sleep quality during pregnancy: A meta-analysis. Sleep Med. Rev..

[30] Ancoli-Israel S., Cole R., Alessi C., Chambers M., Moorcroft W., Pollak C.P. (2003). The Role of Actigraphy in the Study of Sleep and Circadian Rhythms. Sleep.

[31] Lieverse R., Van Someren E.J., Nielen M.M., Uitdehaag B.M., Smit J.H., Hoogendijk W.J. (2011). Bright light treatment in elderly patients with nonseasonal major depressive disorder: a randomized placebo-controlled trial. Arch. Gen. Psychiatry.

[32] Morgenthaler T., Alessi C., Friedman L., Owens J., Kapur V., Boehlecke B., Brown T., Chesson A., Coleman J., Lee-Chiong T. (2007). Practice Parameters for the Use of Actigraphy in the Assessment of Sleep and Sleep Disorders: An Update for 2007. Sleep.

[33] Buysse D.J., Reynolds C.F., Monk T.H., Berman S.R., Kupfer D.J. (1989). The Pittsburgh Sleep Quality Index: a new instrument for psychiatric practice and research. Psychiatry Res..

[34] Cox J.L., Holden J.M., Sagovsky R. (1987). Detection of postnatal depression. Development of the 10-item Edinburgh Postnatal Depression Scale. Br. J. Psychiatry.

[35] Bergink V., Kooistra L., Berg M.P.L.-V.D., Wijnen H., Bunevicius R., Van Baar A., Pop V. (2011). Validation of the Edinburgh Depression Scale during pregnancy. J. Psychosom..

[36] Cohen J. (1988). Statistical Power Analysis for the Behavioral Sciences.

[37] Little R.J.A. (1995). Modeling the Drop-Out Mechanism in Repeated-Measures Studies. J. Am. Stat. Assoc..

[38] Friedman E.S., Davis L.L., Zisook S., Wisniewski S.R., Trivedi M.H., Fava M., Rush A.J., CO-MED Study Team (2012). Baseline depression severity as a predictor of single and combination antidepressant treatment outcome: results from the CO-MED trial. Eur. Neuropsychopharmacol..

[39] Lee K.A., Zaffke M.E., Mcenany G. (2000). Parity and Sleep Patterns During and After Pregnancy. Obstet. Gynecol..

[40] Picchietti D., Winkelman J.W. (2005). Restless Legs Syndrome, Periodic Limb Movements in Sleep, and Depression. Sleep.

